# Starting a career as a psychiatrist

**DOI:** 10.1192/j.eurpsy.2021.214

**Published:** 2021-08-13

**Authors:** N. Sartorius

**Affiliations:** Mental Health, Association for the Improvement of Mental Health Programs, Geneva, Switzerland

## Abstract

**Abstract Body:**

There are several sets of skills first set of skills which psychiatrists should acquire before or as early as possible after starting their career. THe first of those are communication skills – including those of listening, speaking clearly and convincingly, negotiating and writing scientific and other types of documents. A second set of skills are those that will enable psychiatrists to understand and use legal documents and materials. The third set of skills that is likely to be useful are skills necessary to function as a physician. These sets of skills combined with the knowledge of the subject of psychiatry should help in building a career in any of the areas open to psychiatrists.. Yet, more important than any of the skills or bits of knowledge that a candidate psychiatrist should have to build a career and be happy with it are the motivation to do psychiatry and the acceptance of a style of work marked by empathy, willing acceptance of ethical principles of medicine and if at all possible infectious optimism. The above array of skills, knowledge style of work are not easily developed and those educating future psychiatrists should be careful in their selection of trainees and resourceful in the provision of training that will create psychiatrists who can advance the health of their patients as well as their discipline and will have a chance to live a rewarding life.

**Disclosure:**

No significant relationships.

